# Clinical Manifestations of Micronutrient Deficiencies in Short Bowel Syndrome: A Case Report

**DOI:** 10.7759/cureus.37897

**Published:** 2023-04-20

**Authors:** Diana N Rodriguez, Nicole C Ruiz, Steve Qian, Amir Y Kamel

**Affiliations:** 1 Internal Medicine, University of Florida Health Shands Hospital, Gainesville, USA; 2 Gastroenterology and Hepatology, Emory University, Atlanta, USA; 3 Pharmacy, University of Florida Health Shands Hospital, Gainesville, USA

**Keywords:** sma ischemia, citrulline, sma stenting, sma occlusion, total parenteral nutrition (tpn), malnutrition, short bowel syndrome

## Abstract

The etiologies of short bowel syndrome (SBS) can be stratified into congenital or acquired etiologies, with the latter being more prevalent. Small intestinal surgical resection is the most common acquired etiology, employed in settings including mesenteric ischemia, intestinal injury, radiation enteritis, and inflammatory bowel disease (IBD) complicated by internal fistulas. We describe a case of a 55-year-old Caucasian male with a history of idiopathic superior mesenteric artery (SMA) ischemia post-SMA placement complicated by recurrent small bowel obstructions. He presented with SMA stent occlusion and infarction, leaving him with 75 cm of post-duodenal small bowel after emergent surgical resection. He was trialed on enteral nutrition and progressed to parenteral nutrition (PN) after failure to thrive. With intensive counseling, his compliance improved, and he was able to briefly maintain adequate nutrition status with supplemental total parenteral nutrition. After a period of being lost to follow-up, he succumbed to complications from untreated SBS. This case highlights the need for intensive nutritional support for patients with short bowel syndrome and awareness of clinical complications.

## Introduction

Nutritional absorption occurs throughout the small intestine via transport proteins embedded in the villi and microvilli. Most nutritional absorption occurs in the first 100 cm of the jejunum, while vitamin B12 and bile are absorbed primarily in the last 100 cm of the ileum [1]. Short bowel syndrome (SBS) is a malabsorptive disorder defined by the presence of fewer than 180-200 cm of small bowel in situ [2]. Acquired causes include Crohn’s disease, mesenteric ischemia, intestinal injury following a traumatic accident, postsurgical adhesions, and postoperative complications. As a result of the loss of available absorptive surface and transportive areas, patients experience malabsorption of vitamins, minerals, and both micro- and macronutrients. Patients with SBS require a multidisciplinary team for management due to their multifaceted needs. Lifelong follow-up should be led by professionals with an understanding of the anatomy and physiology behind SBS [3]. These patients may additionally be candidates for parenteral nutrition (PN). Current literature has demonstrated that the nonessential amino acid citrulline may serve as an indirect marker for enterocyte mass and intestinal integrity. Due to significant enterocyte loss in SBS, reductions in circulating plasma citrulline have been observed [4].

Both the degree of severity and nutritional deficiency expected in SBS will be determined by factors including the length of the removed or nonfunctioning small bowel and the location of removal or pathological residual bowel [5]. As compared to the large intestine, the small bowel is primarily responsible for nutrient digestion and absorption. Clinical manifestations of SBS include iron, phosphorus, niacin, thiamine, and copper deficiency as a result of duodenal resection, folate deficiency as a result of jejunal resection, and vitamin B12, bile acid, and fat-soluble vitamin malabsorption (A, D, E, and K) as a result of ileal resection. The loss of the ileocecal valve and distal ileum presents as gastric hypersecretion, rapid intestinal transit due to loss of regulatory hormonally driven negative feedback, small intestinal bacterial overgrowth (SIBO), and the loss of zinc, copper, and vitamin D, which are absorbed diffusely throughout the small bowel [6].

## Case presentation

A 55-year-old Caucasian male with a past medical history of superior mesenteric artery (SMA) ischemia of unknown etiology post-SMA stent placement, recurrent small bowel obstructions, hypertension (HTN), and Hodgkin’s lymphoma presented to the hospital with acute exacerbation of his chronic postprandial abdominal pain causing food aversion. His weight was noted to be 57.6 kg, down from 77 kg during his last admission. Pertinent laboratory results on admission including lactic acid may be found in Table 1, remarkable for type 1 lactic acidosis.

**Table 1:** Comprehensive metabolic panel, lactic acid, and weight values, respective to the stage in management Abbreviations: PN: parenteral nutrition, CRP: C-reactive protein, mmol: millimole, L: liter, g: gram, mg: milligram, dL: deciliter, kg: kilogram

	Values pre-massive resection	Values post-massive resection	Values post-PN initiation	Normal reference values
Lactic acid	5.1 mmol/L	1.4 mmol/L	1 mmol/L	0.3-1.5 mmol/L
CRP	164.5 mg/L	17.4 mg/L	15.96 mg/L	<10 mg/L
Albumin	2.4 g/dL	1.9 g/dL	3.5 g/dL	3.5-5.2 g/dL
Prealbumin	3.2 mg/dL	7.1 mg/dL	15 mg/dL	20-40 mg/dL
Total protein	3.6 g/dL	3.5 g/dL	8.5 g/dL	6.4-8.3 g/dL
Phosphate	1.9 mg/dL	6.2 g/dL	3.7 mg/dL	2.7-4.5 mg/dL
Potassium	3.2 mmol/L	2.7 mmol/L	3.7 mmol/L	3.3-5.1 mmol/L
Weight	57.6 kg	48.3 kg	58 kg	-

Vascular and acute care surgery were consulted on admission given computed tomography angiography (CTA) imaging concerning for SMA stent occlusion and multiple dilated small bowel loops. Exploratory laparotomy revealed dense adhesions requiring resection of the ischemic small bowel (SB) and cecum. The vascular surgical service harvested the left femoral vein and successfully performed an antegrade aortomesenteric (SMA) bypass with reversed femoral vein in addition to SMA thromboembolectomy; surgery was completed, and abdominal vacuum-assisted abdominal closure was performed. He had a repeat scheduled exploratory laparotomy that revealed a nonviable edematous right colon and SB; the compromised SB was found to measure 85 cm with a total of 10 cm of SB resected. Post-resection ileocolonic anastomosis was completed, and he was left with 75 cm of SB.

Extensive metabolic and nutrition assessment was performed, and a micronutrient study panel was obtained (Table 2 and Table 3).

**Table 2:** Vitamin, trace element, and iron panel, respective to the stage in management Abbreviations: PN: parenteral nutrition, TIBC: total iron-binding capacity, mg: milligram, L: liter, nmol: nanomole, pg: picogram, mL: milliliter, ng: nanogram, μmol/L: micromole per liter, mcg: microgram, µmol: micromole, μg: microgram, dL: deciliter

Vitamins and trace elements	Values pre-massive resection	Values post-PN initiation	Normal reference values
Vitamin A	0.18 mg/L	0.39 mg/L	0.30-1.20 mg/L
Vitamin B1	287 nmol/L	121 nmol/L	
Vitamin B6	11.2 nmol/L	66.3 nmol/L	20-125 nmol/L
Vitamin B12	865 pg/mL	>1,500 pg/mL	243-894 ng/mL
Folate	>20 ng/mL	>20 ng/mL	>5.8 ng/mL
Vitamin C	10 µmol/L	27 µmol/L	23-114 µmol/L
Vitamin E	7 mg/L	-	5.5-18 mg/L
Vitamin K	-	8.48 nmol/L	0.22-4.88 nmol/L
25-hydroxyvitamin D	8.67 ng/mL	24 ng/mL	20-120 ng/mL
Zinc	-	76 μg/dL	60-120 μg/dL
Iron saturation	7%	26%	20%-55%
Iron serum	10 μg/dL	30 μg/dL	50-160 μg/dL
TIBC	141 mcg/dL	319 mcg/dL	225-319 mcg/dL
Transferrin	111 mg/dL	228 mg/dL	200-360 mg/dL
Ceruloplasmin	10 mg/dL	20 mg/dL	20-60 mg/dL
Copper	79 μg/dL	-	70-140 μg/dL

**Table 3:** Amino acids, respective to the stage in management Abbreviations: PN: parenteral nutrition, µmol: micromole, L: liters

Amino and organic acids	Values pre-massive resection	Values post-massive resection	Values post-PN initiation	Normal reference values
Arginine	44 μmol/L	-	22 μmol/L	40-89 μmol/L
Asparagine	-	11 μmol/L	30 μmol/L	30-90 μmol/L
Carnitine (ester)	-	2 μmol/L	17 μmol/L	50-60 μmol/L
Carnitine (free)	-	21 μmol/L	19 μmol/L	23-59 μmol/L
Carnitine (total)	-	21 μmol/L	36 μmol/L	34-78 μmol/L
Citrulline	-	4 μmol/L	20 μmol/L	15-60 μmol/L
Glutamine	-	413 μmol/L	290 μmol/L	410-750 μmol/L
Glutamic acid	-	124 μmol/L	47 μmol/L	10-120 μmol/L
Glycine	-	385 μmol/L	172 μmol/L	150-450 μmol/L
Homocitrulline	-	<2 μmol/L	<2 μmol/L	0-1.7 μmol/L
Leucine	-	141 μmol/L	79 μmol/L	70-220 μmol/L
Lysine	-	167 μmol/L	91 μmol/L	80-250 μmol/L
Ornithine	-	64 μmol/L	22 μmol/L	20-135 μmol/L
Phenylalanine	-	93 μmol/L	33 μmol/L	50-120 μmol/L
Proline	-	237 μmol/L	111 μmol/L	150-310 μmol/L
Serine	-	129 μmol/L	57 μmol/L	110-250 μmol/L
Taurine	-	49 μmol/L	48 μmol/L	40-200 μmol/L
Tryptophan	-	52 μmol/L	28 μmol/L	25-65 μmol/L
Tyrosine	-	51 μmol/L	31 μmol/L	20-100 μmol/L
Valine	-	517 μmol/L	108 μmol/L	90-370 μmol/L

Due to his poor oral tolerance and newly developed SBS, he was initiated on PN that provided 25 kcal/kg/day, 2 g/kg/day of protein, and 4.2 g/kg/day of glucose based on his ideal body weight of 82 kg. His PN formulation was modified to include 20% intralipid four days per week and standard daily multivitamins 10 mL/day, micronutrients, and trace elements. Prealbumin, albumin, CRP, and weight were followed closely and assessed to ensure adequate repletion (Table 1). Of note, prealbumin was reassessed 10 days after and improved to 9.6 from 3.2 mg/dL. A gastric tube (GT) was placed on day 40 of admission once the patient neared discharge to a long-term care facility. His diet was advanced to clear liquids orally for comfort. His post-hospital discharge course was complicated by abdominal midline wound infection requiring debridement, upper gastrointestinal bleeding with blood draining from GT, sepsis from the infected powerline, candida endocarditis with aortic valve vegetation, and chronic diarrhea complicated by *Clostridium difficile*. Posttreatment of his infection, his hypersecretory state was managed on a maximum dose of Imodium and Lomotil and a tincture of morphine. Unfortunately, he continued to have frequent bowel movements despite the optimization of the multimodal antidiarrheal regimen.

After a few months of being lost to follow-up and self-transitioning off PN, he presented to our emergency department (ED) with fatigue, worsening diarrhea, and failure to thrive in addition to 13.6 kg weight loss. His final admission was complicated by pneumatosis and partial clot within the midportion of the aorta to the SMA graft. He was primarily treated medically rather than surgically given his deconditioning and malnourished status. His hemodynamic status quickly decompensated, leading to septic shock requiring full pressure support in addition to acute hypoxic respiratory failure requiring intubation. After extensive goals of care discussions with his healthcare surrogate, care was withdrawn, and the patient succumbed to his illness.

## Discussion

Micronutrient malnutrition predisposes individuals to impaired immunity, delayed recovery from infection, and increased risk of developing severe infection [7]. Based on the anatomic changes in SBS, patient presenting signs may include electrolyte, micro- and macronutrient deficiencies through malabsorption, dehydration, weight loss, and high output volume loss via diarrhea [8].

We present an SBS patient with idiopathic SMA ischemia requiring stent placement. His clinical course was complicated by SMA stent occlusion, requiring thrombectomy, SMA bypass, and multiple surgical revisions resulting in the retention of only 75 cm of functional bowel. Although he was trialed on evidence-based regimens for his diarrhea and malabsorptive status, as stated in the case description, he required parenteral nutrition to augment his oral intake given ongoing nutritional deficits in addition to failure to thrive.

Extensive evaluation by our nutrition specialists determined him to have significant deficiencies prior to initiation of PN, including carnitine, vitamin A, vitamin B6, vitamin C, 25-hydroxyvitamin D, and iron. Specifically, his vitamin B6 was found to be decreased at 11.2 nmol/L pre-massive resection and increased to 66.3 nmol/L post-PN initiation. Based on literature regarding the anti-inflammatory effect of vitamin B6, his pre-resection state with low vitamin B6 may have contributed to his pro-inflammatory state [5]. His low vitamin D level (Table 3) likely further contributed to both a pro-inflammatory state and vulnerability to acquiring infections [9].

Further supporting his pro-inflammatory state pre-resection was his anemia of chronic disease and total low iron saturation, a well-known phenomenon of chronic inflammation [10]. In addition, amino acid assay revealed low asparagine, glutamine, taurine, tyrosine, and citrulline, indicating prolonged severe malnutrition and inadequate absorptive capacity. Through enteral nutrition, the aforementioned micro- and macronutrients in addition to vitamins and minerals were aggressively replenished. After an aggressive PN regimen, our patient had a significant increase in weight, in addition to improvement of several nutrition parameters and repletion of micronutrient deficiencies (Tables 1-3).

Regarding guideline-directed recommendations for the nutritional management of SBS patients, Parrish et al. [3] suggested that the management of SBS requires tailoring therapy based on the resected bowel, remaining anatomy, and individualized psychosocial characteristics. Multidisciplinary management of SBS with PN was reported to be associated with patient survival of 86% and 75% at two and five years, respectively [11]. Guidelines for the management of patients with SBS recommend a personalized clinical assessment including water, sodium, magnesium, and nutritional status in the context of the residual remaining small bowel length [12]. The European Society for Clinical Nutrition and Metabolism (ESPEN) practice guidelines regarding clinical nutrition in chronic intestinal failure highlight specifics regarding the management of home parenteral nutrition, components of parenteral nutrition, and both dietary and pharmacological agents to aid in intestinal rehabilitation, specifically, recommendations regarding macronutrients, fluids, electrolytes, vitamins, trace elements, and amino acids. The ESPEN recommends that patients initially discharged on PN be closely monitored every few days with prolongation of the interval between follow-up time to become every week and eventually every month [12]. Table 4 is a summary of ESPRN recommendations for adult patients with SBS.

**Table 4:** ESPEN guidelines (2021) ESPEN: European Society for Clinical Nutrition and Metabolism, SBS: short bowel syndrome, PN: parenteral nutrition, EFA: essential fatty acids, Source: [12]

SBS				
Personalized multidisciplinary metabolic and nutrition assessment				
PN recommended components	Dietary intestinal rehabilitation	Antisecretory agents	Antimotility agents	Bile acids and bile acid binders
Adherence to a high-carbohydrate and low-fat diet is recommended. Limiting long-chain fatty acids that reduce transit time and exacerbate the reduction of water and electrolyte absorption is recommended. Supplementation of PN with lipid emulsion containing EFA to prevent EFA deficiency is recommended. Regular monitoring of fluid balance, electrolytes, mineral balance, and acid-base status is recommended. Baseline measurement of vitamin and trace element concentrations with yearly evaluation is recommended. The routine addition of amino acids is not recommended given current data lacks supporting evidence of improved patient outcomes.	SBS patients should be advised to partake in dietary counseling and encouraged to consume regular food items. The addition of glutamine, probiotics, or other supplemental nutrients to the diet is not recommended. The addition of soluble fiber is not recommended to enhance intestinal absorption. Lactulose is suggested not to be excluded from the diet of SBS patients unless previous intolerance has been demonstrated. Optimal blood glucose control during PN given the high rate of adverse outcomes in patients with hyperglycemia.	The use of H2 receptor antagonists and proton pump inhibitors in reducing the excretion of sodium and the fecal wet weight is recommended. Patients on proton pump inhibitors require monitoring for Clostridium difficile and hypomagnesemia. In the SBS population, specifically post-intestinal resection, the use of octreotide is recommended in those who have failed conventional treatment of high output state.	The use of loperamide is recommended to decrease fecal sodium loss and reduce wet weight. Optimization of non-opiate drugs including loperamide and atropine is recommended to decrease exposure to addictive agents. Atropine requires careful use in the elderly population. Opiate alternatives include codeine and tincture of opium and may be considered in patients who fail management with loperamide or atropine.	Resection of more than 100 cm of the terminal ileum affects bile acid reabsorption. The use of bile acid sequestrants is not routinely recommended as they may further reduce bile and worsen steatorrhea and loss of fat-soluble vitamins. Bile acid binders may be considered in SBS patients who have intact colons and have failed previous therapy.

Table 3 highlights several vitamin and trace element deficiencies our patient experienced. It is important to recognize the clinical impact of these deficiencies potentially produced in this patient. Our patient experienced over 10 admissions to the emergency department over the span of a few months, several of these with diagnosis codes including sepsis, severe sepsis, and septic shock. Sommer et al. [13] suggest a link between vitamin A deficiency and susceptibility to severe infection, posing a plausible component to this patient’s severity of infections. Both Maxfield et al. [14] and Maxfield et al. [15] agree that vitamin C and zinc deficiency may contribute to the decreased physiological response to infections and result in suboptimal wound repair, these potentially contributing to our patient as recurrent decubitus ulcers.

In summary, patients who develop SBS have variability in their distinct degree of surgical resection and degree of malabsorptive inability that ensues. Outcomes are multifactorial and are affected by the degree of the available absorptive surface area, the quality of the remaining bowel, and superimposed conditions that may alter the remaining architecture. Supporting patients through their metabolic and nutritional needs, as evidenced by our patient, can result in optimal patient management and control of their underlying disease process. Although the care of SBS patients is complex and multifaceted, as evidenced by our patient, close management by a multidisciplinary team is essential.

## Conclusions

Patients with acquired SBS require management by an interprofessional team including a physician with specialized training in gastroenterology. The primary driver of their extensive macronutrient, micronutrient, electrolyte, fluid, and amino acid disturbances is intestinal malabsorption that results from a decrease in absorptive tissue and rapid transit time. By virtue of this case report, we hope to encourage close surveillance of SBS patients and bring further awareness of the multisystem complications that arise both in the early course of the disease and from medically undermanaged SBS.
